# The Diversity and Spatiotemporally Evolutionary Dynamic of Atypical Porcine Pestivirus in China

**DOI:** 10.3389/fmicb.2022.937918

**Published:** 2022-06-24

**Authors:** Hailong Ma, Wentao Li, Mengjia Zhang, Zhengxin Yang, Lili Lin, Ahmed H. Ghonaim, Qigai He

**Affiliations:** ^1^State Key Laboratory of Agricultural Microbiology, College of Veterinary Medicine, Huazhong Agricultural University, Wuhan, China; ^2^Key Laboratory of Preventive Veterinary Medicine in Hubei Province, The Cooperative Innovation Center for Sustainable Pig Production, Wuhan, China; ^3^Desert Research Center, Cairo, Egypt

**Keywords:** diversity, spatiotemporal evolution, selection pressure, geography-driven adaptation, China, APPV

## Abstract

The presence of congenital tremor (CT) type A-II in newborn piglets, caused by atypical porcine pestivirus (APPV), has been a focus since 2016. However, the source, evolutionary history, and transmission pattern of APPV in China remain poorly understood. In this study, we undertook phylogenetic analyses based on available complete E2 gene sequences along with 98 newly sequenced E2 genes between 2016 and 2020 in China within the context of global genetic diversity. The phylogenies revealed four distinct lineages of APPV, and interestingly, all lineages could be detected in China with the greatest diversity. Bayesian phylogenetic analyses showed that the E2 gene evolves at a mean rate of 1.22 × 10^−3^ (8.54 × 10^−4^-1.60 × 10^−3^) substitutions/site/year. The most recent common ancestor for APPVs is dated to 1886 (1837–1924) CE, somewhat earlier than the documented emergence of CT (1922 CE). Our phylogeographic analyses suggested that the APPV population possibly originated in the Netherlands, a country with developed livestock husbandry, and was introduced into China during the period 1837–2010. Guangdong, as a primary seeding population together with Central and Southwest China as epidemic linkers, was responsible for the dispersal of APPVs in China. The transmission pattern of “China lineages” (lineage 3 and lineage 4) presented a “south to north” movement tendency, which was likely associated with the implementation of strict environmental policy in China since 2000. Reconstruction of demographic history showed that APPV population size experienced multiple changes, which correlated well with the dynamic of the number of pigs in the past decades in China. Besides, positively selected pressure and geography-driven adaptation were supposed to be key factors for the diversification of APPV lineages. Our findings provide comprehensive insights into the diversity and spatiotemporal dynamic of APPV in China.

## Introduction

Emerging viral diseases in pigs have increased in the past decades and pose a serious risk to the pig sector globally. In 2015, a novel porcine pestivirus, termed atypical porcine pestivirus (APPV), was first discovered during a metagenomic sequencing project in the USA (Hause et al., 2015). Later, an experimental inoculation study conducted in pregnant sows demonstrated that APPV was associated with congenital tremor (CT) type A-II in newborn pigs (Arruda et al., 2016). So far, APPV-associated CT has been reported in multiple continents, including Europe, America, and Asia (Riedel et al., 2021). Due to the lack of proof-reading exonuclease activity of RNA polymerase, APPV strains worldwide have genetically high sequence variability (Postel et al., 2017). Unsurprisingly, the highest diversity of APPVs to date was detected in China, which is an important pig-raising and pig-trading country (Riedel et al., 2021).

Atypical porcine pestivirus is an enveloped and positive-stranded RNA virus. Within the genus *Pestivirus*, APPVs belong to the species *Pestivirus K* and are closely related to pestiviruses isolated from bats (Wu et al., 2018). The viral genome is about 11.0 kb in size and comprises a single open reading frame (ORF). The ORF encodes a continuous polyprotein, which is processed into 12 mature proteins, including three envelope proteins, including Erns, E1, and E2 (Simmonds et al., 2017). As a critical structural glycoprotein, E2 has been recognized as the receptor-binding protein for CSFV and BVDV (Simmonds et al., 2017). More importantly, E2 is the immunodominant antigen to independently induce protective antibodies and is able to exhibit the greatest amount of diversity in pestiviruses (Simmonds et al., 2017). Consequently, the full-length E2 gene often acts as a phylogenetic target to address viral evolutionary relationships (Postel et al., 2012; Rios et al., 2017). Recently, titers of neutralizing antibodies in APPV-infected piglets were proved to correlate with the level of E2-specific antibodies, suggesting that the E2 gene is likely the immunodominant antigen for APPV (Cagatay et al., 2018).

Phylogenetically, based on the different genomic regions, APPVs could be clustered into three major lineages with a high level of genetic variation (Guo et al., 2020; Yuan and Wang, 2021; Yuan et al., 2021). Compared to lineage 2 and lineage 3, both of which were only found in China, strains from lineage 1 seemed to be prevalent worldwide and displayed higher genetic variation (Guo et al., 2020). Limited recombination events could be detected between lineage 2 and lineage 3 or within lineage 1 (Guo et al., 2020). Possibly due to an adaptability advantage over recombinants, the non-recombinants dominate in various regions of the world. Although the role of genome recombination in the evolution of APPV remains unclear, it is confirmed that recombination could allow viruses to rapidly acquire adaptive capability in hosts and constantly evolve with increasing genetic diversity.

The Bayesian phylodynamic inference is a well-developed approach in the field of virology, and is used widely for estimating the origin of epidemics and tracking the spatial dissemination of viruses (Pybus and Rambaut, 2009). This approach has been accepted in studying the evolutionary dynamics of influenza viruses and other RNA viruses from animals (Su et al., 2015; Sun et al., 2019). So far, some aspects of epidemiology in APPV have been studied intensively (Dall Agnol et al., 2020; Riedel et al., 2021). However, owing to a lack of sequence data, a few studies focus on the timescale and evolutionary dynamic of APPV. Only a recent study based on 58 complete genomic sequences showed that APPV evolved at a mean rate of 1.37 × 10^−4^ substitutions/site/year, and the most recent common ancestor was dated to 1700 CE (Shi et al., 2021), but detailed works on temporal signal test and best-fitting model for nucleotide substitution, clock, and tree prior were not estimated, leaving uncertainty for the inference. Additionally, there remains a poor understanding of the migration pathways of APPV across spatial scales in China.

In this study, we undertook phylogenetic analyses of available E2 gene sequences sampled from Chinese cases along with additional 98 newly sequenced E2 genes within the context of global genetic diversity to characterize the epidemiology of APPV in China. Here, we provide insights into the temporally and spatially evolutionary dynamic of APPV. International introductions are expected to play an important role in seeding transmission pathways in China. In addition, the differences in selection pressure acting on APPV lineages were depicted.

## Materials and Methods

### Sample Collection, Amplification, and Sequencing

Piglet CT surveillance was primarily conducted in China (Supplementary Table S1). Serum and tissue samples were collected from neonatal pigs with CT-like symptoms on pig farms. All samples were treated as described previously and were negative for PRV, PRRSV, CSFV, and PCV2 (Ma et al., 2021). APPV-positive samples were used for E2 gene sequencing (Supplementary Table S1).

### Recombination and Phylogenetic Analyses

All nucleotide sequences were aligned using MAFFT (Katoh and Standley, 2013). The full-length E2 sequence alignment was used to detect recombination signals using the neighbor-net method in SplitsTree 4.13.1 (Huson, 1998). Pairwise Homoplasy Index (PHI) was calculated with split-decomposition network analysis. Recombination events were further identified using a suite of methods, including RDP, GENECONV, BootScan, MaxChi, Chimaera, SiScan, and 3Seq in RDP 4.95 (Martin et al., 2015), which was supported by at least four methods with strict criteria (*P* < 10^−6^ and recombination score > 0.6). Recombination-free E2 datasets were generated, and the best model of nucleotide evolution was identified using jModelTest 2.1.10 (Darriba et al., 2012). A maximum-likelihood phylogeny was reconstructed using the GTR+G+I model in MEGA 7.0.21 (Kumar et al., 2016). Branch robustness was assessed using bootstrapping method with 1,000 replicates. The original tree was visualized in FigTree 1.4.4 (http://tree.bio.ed.ac.uk/software). Genetic divergence within and between APPV lineages was calculated using the p-distance model in MEGA 7.0.21 (Kumar et al., 2016).

### Tests for Temporal Signal

A preliminary analysis of the temporal signal was conducted based on a regression method of root-to-tip distances against sampling dates in TempEst 1.5.3 (Rambaut et al., 2016). For this analysis, a maximum-likelihood tree was reconstructed using IQ-Tree (Nguyen et al., 2015). To exclude the impacts of highly genetic structure on temporal signal test and molecular dating, a Mantel test to assess the confounding between genetic distances and temporal distances was performed with 1,000 permutations (Murray et al., 2016). Then, a date-randomization test using clustered permutation to evaluate the temporal signal by randomizing the sampling dates was conducted (Duchêne et al., 2015). The mean substitution rates estimated were visualized in the TipDatingBEAST package (Rieux and Khatchikian, 2017). Besides, Bayesian Evaluation of Temporal Signal (BETS), a fully Bayesian model testing strategy, was adopted to confirm the temporal signal (Duchene et al., 2020). This approach relies on the comparison of the fitness between the heterochronous model and isochronous model using Marginal Likelihood (log) Estimation (MLE), which was estimated by stepping stone sampling (Baele et al., 2012).

### Temporal Dynamic of APPV

To reconstruct the evolutionary rate and time scale of APPV, a Bayesian inference based on the Markov chain Monte Carlo (MCMC) framework was implemented in BEAST 1.10.4 (Suchard et al., 2018). For our dataset, the best-fit model of nucleotide substitution (GTR+G4) was selected using ModelFinder (Nguyen et al., 2015; Zhang et al., 2020). We compared the MLEs of different combinations between two clock models (strict clock and uncorrelated lognormal relaxed clock) and four coalescent tree priors (constant size, exponential growth, Bayesian skyline, and Bayesian skygrid) using stepping stone sampling to determine the best-fit model (Baele et al., 2012). Two independent MCMC chains for 400 million steps were performed with sampling every 10,000 steps and were combined with a burn-in of 10%. Convergence of runs was inspected in Tracer 1.7.1 (ESS > 200) (Rambaut et al., 2018). The skygrid line plots and maximum clade credibility trees were, respectively, visualized in Tracer 1.7.1 and FigTree 1.4.4.

### Discrete Phylogeography Analyses

According to the sampling locations, we divided E2 sequences into seven geographic regions: Austria Region (AuR, *n* = 1), Germany Region (GerR, *n* = 4), South Korea Region (SKoR, *n* = 5), Netherlands Region (NeR, *n* = 9), Switzerland Region (SwR, *n* = 7), USA Region (AmR, *n* = 4), and China Region (ChnR, *n* = 124). To trace domestic migration pathways of APPV, we divided mainland China into six regions based on the locations of sampling provinces (Supplementary Figure S1): North China Region (NCR, *n* = 7), Northeast China Region (NeCR, *n* = 5), Central China Region (CCR, *n* = 15), East China Region (ECR, *n* = 19), Southwest China Region (SwCR, *n* = 12), and South China Region (SCR, *n* = 66). To mitigate potential sampling bias, we divided sequences from SCR into Guangdong Region (GDR, *n* = 38) and Guangxi Region (GXR, *n* = 28). We deleted Austria Region where a single sequence was found (AUT-2016_C). Finally, a dataset consisting of 153 E2 sequences with 12 geographic locations across six countries was assembled.

To understand the spatial dynamic of APPV, phylogeographic analyses were performed in BEAST 1.10.4 (Minin and Suchard, 2008). An asymmetric substitution model for a discrete trait with Bayesian stochastic search variable selection (BSSVS) was applied to infer asymmetric migration rates between pairwise regions. Two independent analyses for 400 million steps were performed as above. The diffusions between discrete regions were estimated in SPREAD3 0.9.7 (Bielejec et al., 2016). Significant migration pathways were summarized based on the combination of both BF > 3 and the corresponding node posterior probability > 0.50. The significant degree is defined as follows: BF > 100 indicates decisive, 30 < BF <100 indicates very strong support, 10 < BF <30 indicates strong support, and 3 < BF <10 indicates substantial support (Jeffreys, 1961). In addition, we estimated all the transitions (Markov Jumps) between regions using the asymmetric migration pattern (Minin and Suchard, 2008), and plotted the total number (all possible) of state counts for the diffusion in and out of each region. We also estimated the time of migration events (Markov Rewards) spent in the different regions based on the reconstruction of ancestral states.

### Phylogeny–Geography Association Analyses

To assess the association between phylogeny and location compartmentalization, three statistics were employed in BaTS (Parker et al., 2008). The values of association index (AI), parsimony score (PS), and monophyletic clade (MC) size statistics were calculated from the posterior trees above. This method accounts for phylogenetic uncertainty investigating phylogeny–location association, with 1,000 random permutations of geographic locations to estimate an expected (null) distribution for each statistic. Low AI and PS and high MC values suggest a strong phylogeny–geography correlation. The ratios of the observed to null mean AI and PS were calculated as a measure to assess the strength of the association between phylogeny and location.

### Natural Selection

To compare the selection pressures on APPV lineages, the ratios of non-synonymous to synonymous (*d*_N_/*d*_S_) substitution rates for different lineages were estimated using the branch model in EasyCodeML (Gao et al., 2019). The likelihood ratio test (LRT) was used to screen the best model with an X2-distribution. Then, MEME and FEL methods (*p* < 0.05) in Datamonkey (Kosakovsky Pond and Frost, 2005; Murrell et al., 2012) and the site model in EasyCodeML (Gao et al., 2019) were used to detect the positively selected sites. For the site model, M8 + BEB method was applied (posterior probability > 0.9). The tertiary structure of the E2 monomer was built using I-TASSER (Yang and Zhang, 2015). C-score was used to estimate the model quality [−5, 2]. A TM-score >0.5 indicated a model with correct topology. All positive sites were mapped with PyMOL (v2.3.0).

## Results

### Sampling and Sequencing of APPV in China

**Figure 1:**
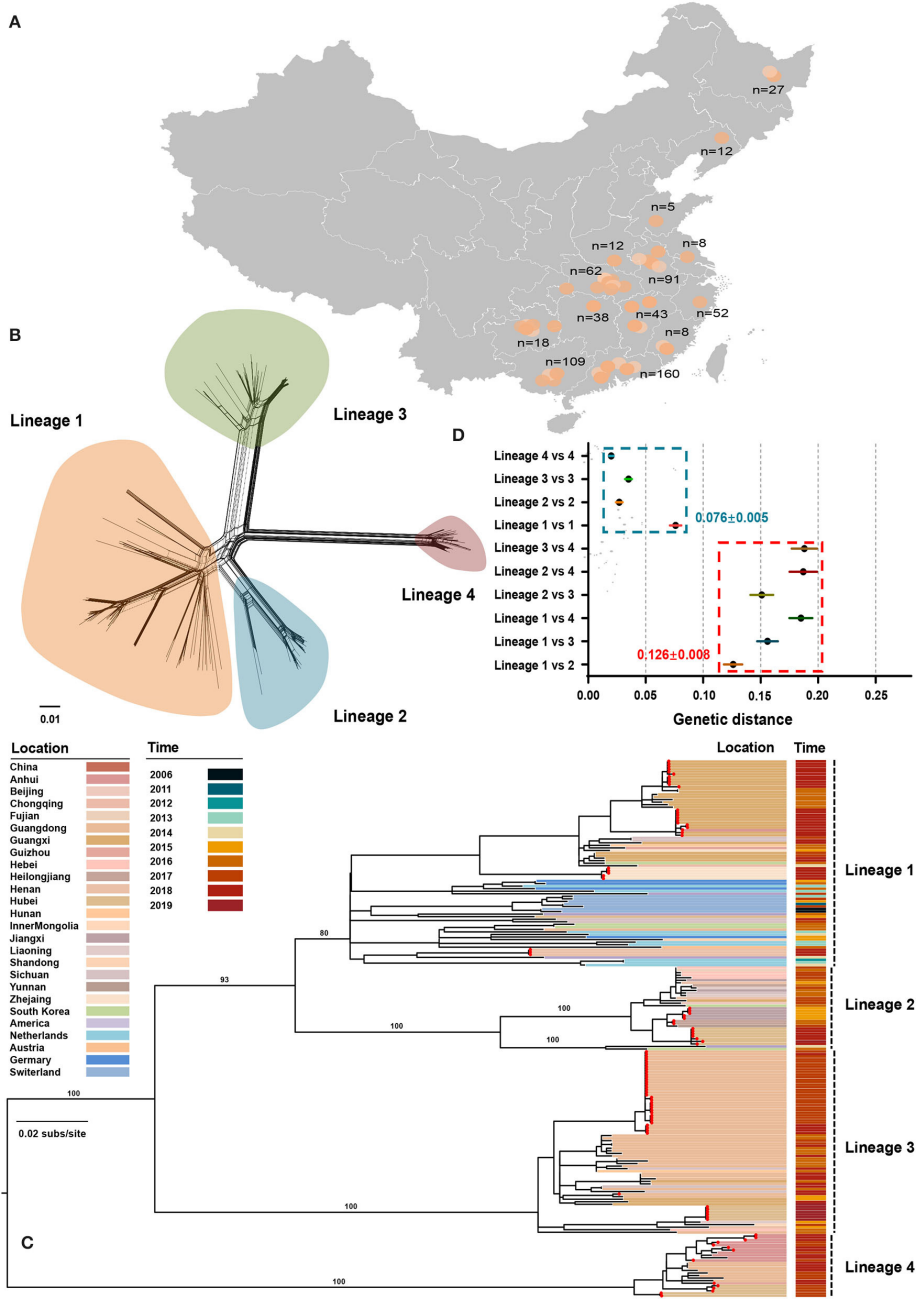
Phylogenetic relationships between atypical porcine pestivirus. **(A)** Map showing the sampling locations selected in this study. **(B)** Splits network analysis of 211 E2 sequences. The pairwise homoplasy index test did not find significant evidence for recombination (*p* = 0.45). **(C)** Maximum-likelihood phylogeny based on 211 E2 sequences with sampling locations and dates from 2006 to 2019. Red dots indicate the strains sequenced in this study. The scale bar is given in substitutions/site. **(D)** Evolutionary divergence analysis between and within lineages.

From July 2016 to September 2020, 645 tissue samples were collected from neonatal pigs with CT-like symptoms, of which 91.01% were obtained between July 2016 and July 2018 (Supplementary Table S1). The number of clinical samples decreased sharply in late 2018, which could be attributed to the emergence of African swine fever in China (Zhou et al., 2018). All samples were collected across 14 provinces of China with a considerable diversity of sampling locations (Figure 1A). APPV-positive samples were used for the amplification of the E2 gene with a nested PCR method (Supplementary Table S1). Finally, 98 complete E2 genes were obtained and were deposited into the GenBank database (Supplementary Table S2). In addition, 135 published E2 sequences were downloaded from GenBank and submitted before March 2021. After discarding low-quality sequences with degenerate bases, incomplete coding regions, unknown sampling dates and locations, and sequences from wild pigs, a dataset containing 211 E2 sequences from seven countries between 2006 and 2020 was generated (Supplementary Table S2). Using SplitsTree program, split network analysis did not show evidence of reticulate topology suggesting that APPVs have not experienced recombination in the E2 region, which was consistently supported by the pairwise homoplasy index test (Figure 1B, *p* = 0.45). Likewise, no significant signals of recombination were detected by the RDP package. Therefore, the dataset of 211 full-length E2 sequences was used for performing phylogenetic analyses.

### The Diversity of APPV Lineage

The maximum-likelihood phylogeny revealed four distinct lineages (Figure 1C). Lineage 1 contained 81 strains across seven countries, including China, South Korea, the USA, Netherlands, Germany, Switzerland, and Austria, of which 54 were originated from 11 provinces of China. The unresolved topology of this lineage indicated a complicated relationship during evolution. Considering that strains of lineage 1 covered more countries and had the greatest nucleotide diversity (Figure 1D), lineage 1 is probably the earliest APPV lineage. Besides, both earliest strains that were detected in Switzerland in 2006 also belonged to this lineage. Lineage 2 contained 33 strains from China, the USA, and South Korea. One strain from the USA and two from South Korea together formed a sister sub-lineage to the remaining China strains with high support. Lineage 2 could circulate in 11 provinces of China, representing great the diversity of prevalent locations. In contrast, lineage 3 and lineage 4 were only reported in China, often regarded as “Chinese Lineage.” Seventy-three strains from lineage 3 formed a strongly supported MC containing strains from nine provinces of China. Similarly, lineage 4 included 25 strains from four provinces of China. Taken together, these results suggested that four APPV lineages co-exist in Chinese pig populations, and each lineage could circulate in multiple provinces. On the other hand, we found that multiple lineages could circulate in a province showing lineage diversity of APPV in this region (Supplementary Table S3). For example, APPV lineage 1/2/3/4 could co-exist in Hubei province, a region in Central China. The potential effect of regional co-prevalence of multiple APPV lineages on susceptible animals was worthy of further concern. Additionally, evolutionary divergence analysis revealed that lineage 1 and lineage 2 showed the least genetic distance (0.126 ± 0.008) between lineages, which was higher than the largest genetic distance (0.076 ± 0.005) within lineages, suggesting a cutoff value of genetic distance (0.126 ± 0.008) for defining APPV types (Figure 1D).

### Evolutionary Rate and Timescale of APPV

A dataset containing 154 E2 sequences without identical sequences was generated to assess the temporal structure (Supplementary Table S2). Using TempEst, the linear regression plot revealed a mild molecular-clock-like signal for our data (Supplementary Figure S2, *r* = 0.43). To exclude the potential impact of highly genetic structure on the temporal signal test, a Mantel test was performed and showed no evidence of confounding of temporal and genetic structures (Supplementary Figure S3, *p* = 0.40). Meanwhile, our data passed criterion 2 of the clustered date-randomization test that the 95% confidence intervals of the mean substitution rate estimated from 154 E2 sequences did not fall within the 95% confidence intervals of the rate estimates obtained from 20 replicates with cluster-permuted sampling dates (Supplementary Figure S4). This evidently demonstrated sufficient temporal signal in our dataset, which was further confirmed by the Bayesian Evaluation of Temporal Signal (Supplementary Table S4, log BF = 49.26).

**Figure 2:**
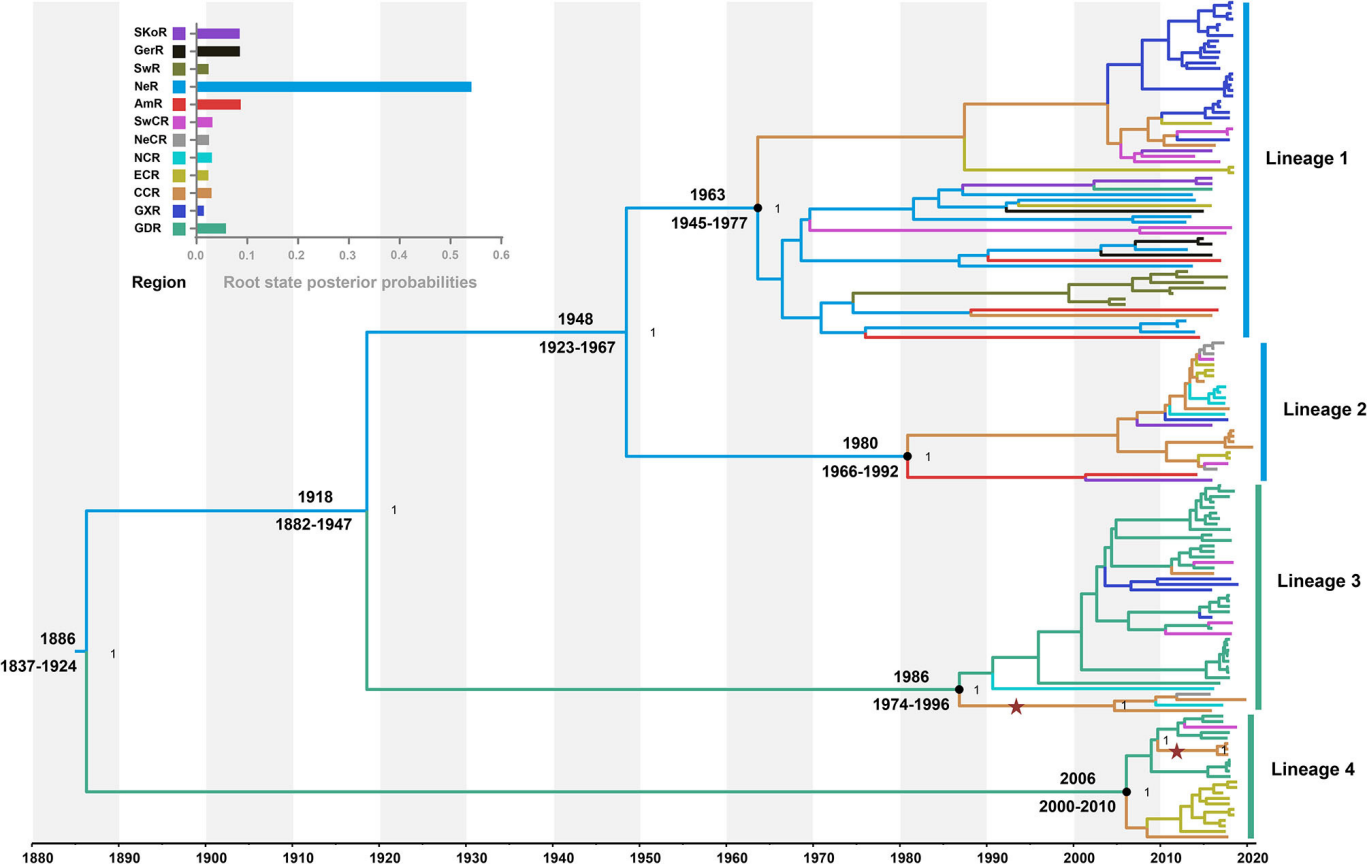
Time-scaled maximum clade credibility tree inferred from E2 gene dataset. Black dots indicate the most recent common ancestors for lineages 1, 2, 3, and 4 with support of posterior probability = 1.00. Branch colors denote inferred region states. The root state posterior probabilities of the geographic regions are shown in the inset panel. SKoR, South Korea Region; GerR, Germany Region; SwR, Switzerland Region; NeR, Netherlands Region; AmR, USA Region; SwCR, Southwest China Region; NeCR, Northeast China Region; NCR, North China Region; ECR, East China Region; CCR, Central China Region; GXR, Guangxi Region; GDR, Guangdong Region. Introduction of atypical porcine pestivirus into CCR from GDR is marked with stars.

The model combination of Bayesian skygrid coalescent tree prior and strict molecular clock provided the best fit for our dataset (Supplementary Table S5). The Bayesian estimated mean substitution rate was 1.22 × 10^−3^ (95% confidence interval: 8.54 × 10^−4^-1.60 × 10^−3^) substitutions/site/year. The time-scaled maximum clade credibility (MCC) tree showed four MCs, respectively, corresponding to the lineages 1, 2, 3, and 4 from ML phylogeny (Figure 2). Four APPV lineages shared a common ancestor in 1886 (95% confidence interval: 1837–1924) CE. The most recent common ancestor for lineage 1, lineage 2, lineage 3, and lineage 4 was 1963 (1945–1977) CE, 1980 (1966–1992) CE, 1986 (1974–1996) CE, and 2006 (2000–2010) CE, respectively.

### The Pattern of Geography Structure of APPV

To understand the potential association between phylogeny and geographic traits of APPV, we analyzed the E2 dataset with sampling dates and regions using BaTS. The tests of AI and PS statistics strongly supported an association between phylogeny and geographic trait, although the time-scaled MCC tree seemed to not mirror an obvious geographic structure (Table 1; AI, *p* < 0.001; PS, *p* < 0.001). Based on the MC size statistic, APPV population subdivision was observed in 10 geographic regions, except USA Region (AmR) and Southwest China Region (SwCR), indicating significant spatial structure in the diversification of APPV. We calculated index ratios of the observed to the expected mean values for AI and PS to assess the strength of the association between phylogeny and geographic traits. The ratios of 0.27 (95% confidence interval: 0.22–0.34) for AI and 0.48 (0.44–0.51) for PS among the geographic regions confirmed the important role of geographic structure in the evolution of APPV.

**Table 1:** Phylogeny-trait correlation analysis for the geographic structure of APPV using BaTS method.

**Statistic**	**Strains**	**Index ratio (95% HPD CIs)**	**Observed mean (95% HPD CIs)**	**Null mean (95% HPD CIs)**	***P*-value**
Association index		0.27 (0.22–0.34)	4.25 (3.62–4.92)	15.64 (14.59–16.70)	<0.001
Parsimony score		0.48 (0.44–0.51)	49.06 (47.00–51.00)	102.42 (99.11–105.70)	<0.001
**Monophyletic clade (MC) size**
MC (ECR)	19		10.00 (10.00–10.00)	1.55 (1.00–2.01)	<0.01
MC (GDR)	38		9.44 (9.00–11.00)	2.37 (1.96–3.26)	<0.01
MC (GXR)	28		18.00 (18.00–18.00)	1.97 (1.20–3.00)	<0.01
MC (SwCR)	12		2.00 (2.00–2.00)	1.35 (1.00–2.00)	0.15
MC (NeCR)	5		2.18 (2.00–3.00)	1.07 (1.00–1.87)	0.04
MC (CCR)	15		4.00 (4.00–4.00)	1.41 (1.00–2.04)	<0.01
MC (AmR)	4		1.00 (1.00–1.00)	1.03 (1.00–1.07)	1.00
MC (NeR)	9		3.00 (3.00–3.00)	1.14 (1.00–2.00)	<0.01
MC (GerR)	4		2.00 (2.00–2.00)	1.03 (1.00–1.06)	0.04
MC (NCR)	7		2.04 (1.00–4.00)	1.11 (1.00–1.99)	0.03
MC (SKoR)	5		2.00 (2.00–2.00)	1.04 (1.00–1.15)	0.02
MC (SwR)	7		7.00 (7.00–7.00)	1.09 (1.00–1.91)	<0.01

*BaTS accounts for uncertainty from phylogenetic error and provides a statistical significance test for the null hypothesis that traits are associated randomly with phylogeny tips.*

*AI, Association index; PS, Parsimony score; MC, Monophyletic clade; Index ratio, observed mean/null mean; HPD CIs, highest posterior density confidence intervals; P-value, statistical significance*.

### Migration Pattern of APPV

**Figure 3:**
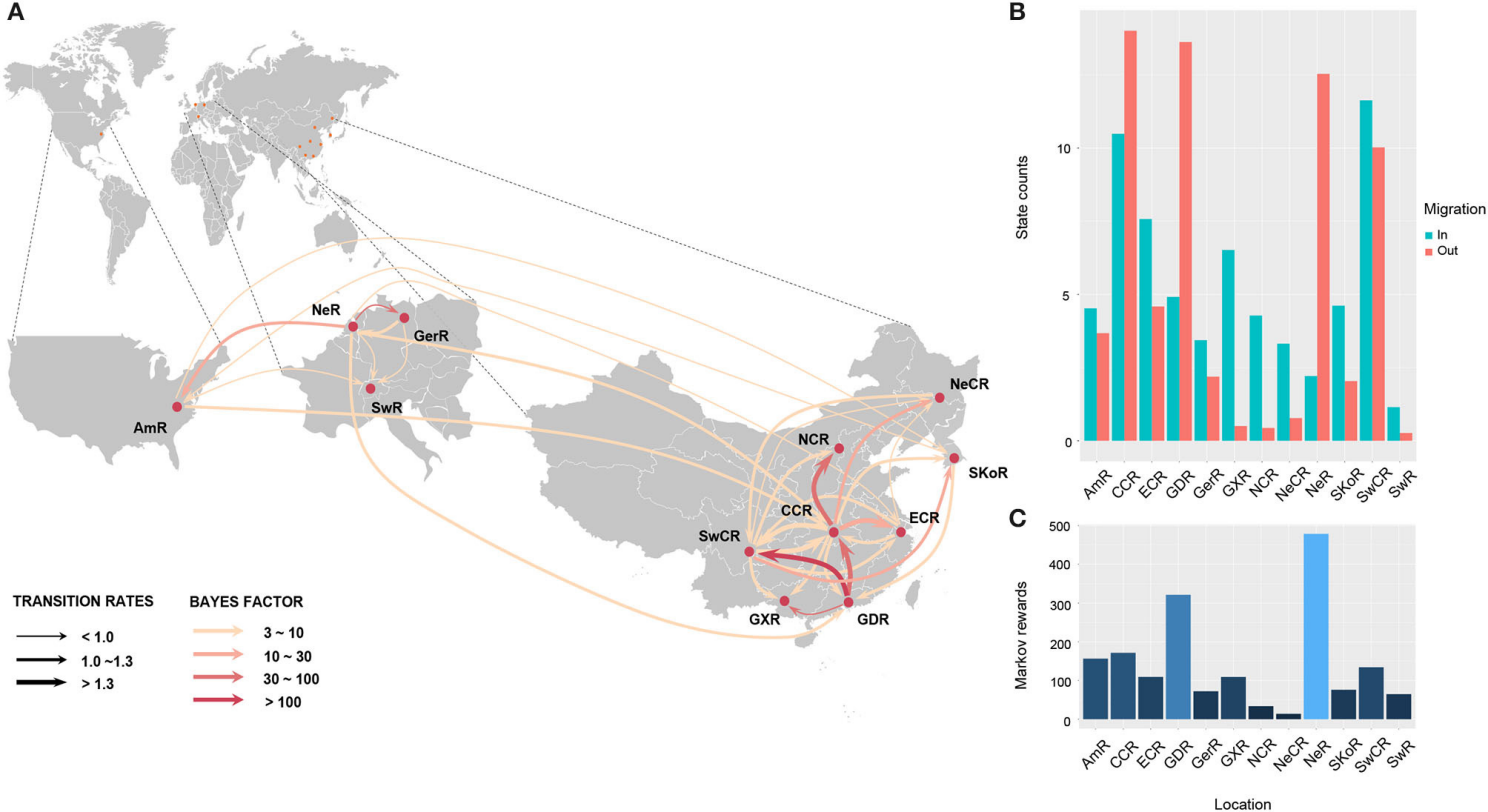
Spatial migration of atypical porcine pestivirus. Supported migration pathways with different transition rates **(A)**, and both histograms of the total number of state counts for the migrations **(B)** and the time of transitions spent in each region **(C)** inferred from the E2 gene dataset. Gradual solid arrows, respectively, indicate decisively supported diffusions (BF > 100), very strongly supported diffusions (30 < BF <100), strongly supported diffusions (10 < BF <30), and supported diffusions (3 < BF <10). SKoR, South Korea Region; GerR, Germany Region; SwR, Switzerland Region; NeR, Netherlands Region; AmR, USA Region; SwCR, Southwest China Region; NeCR, Northeast China Region; NCR, North China Region; ECR, East China Region; CCR, Central China Region; GXR, Guangxi Region; GDR, Guangdong Region.

To understand the potential introduction events and the migration pattern of APPV in China, a Bayesian discrete phylogeographic framework was applied to reconstruct the past spatial dynamics for 12 geographic regions, including seven domestic regions (Supplementary Figure S1) and five from other countries. The results placed the ancestral root location of APPVs in the Netherlands, and the main trunk of the MCC tree was also characterized by an origin in the Netherlands (Figure 2). At the country level, 15 significant migration pathways of APPV across six countries were supported by our phylogeographic analyses, of which six originated from the Netherlands to other four countries (4/5), including Switzerland, the USA, China, and Germany (Figure 3A, Supplementary Table S6). The population of APPV from the Netherlands possibly acted as the main source for the epidemics in other countries. This was supported by state counts (that is, the total number of transitions of geographical locations), with migration from the Netherlands being greater than from other countries (Figure 3B). Although slightly higher state counts could be indicated in Central China and Guangdong regions, no supported migrations from China to other countries were observed, except in South Korea (Figure 3A, Supplementary Table S6). We plotted the Markov jump counts (that is, all the transitions between states) for the Netherlands, demonstrating multiple migrations from the Netherlands to other countries with a marked outward bias (Figure 3B, Supplementary Figure S5). Additionally, we estimated the Markov rewards (that is, the time of transitions spent in a region) for the trunk regions (Figure 3C). The Netherlands occupied the phylogenetic tree trunk with the highest reward time of 478.56, further suggesting the significant role of the Netherlands as a seeding population during the transmission of APPV.

In China, 18 significant migrations across seven regions were indicated with mean rates from 0.92 to 1.70. Six migrations originated from Central China to East China, Guangdong, Guangxi, North China, Northeast China, and Southwest China, which covered all sampled regions, and three from Guangdong to Central China, Guangxi, and Southwest China (Figure 3A, Supplementary Table S6). High mean rates for migrations were observed from Guangdong to Southwest China and from Central China to East China (Supplementary Table S6, 1.75 and 1.53, respectively). State counts reflected this dynamic with outward migration from Central China and Guangdong (Figure 3B). However, the mean migration rate and Markov jump counts showed apparent migrations out of Guangdong to other locations (including migration from Guangdong to Central China was very strongly supported by BF = 45.8) but nearly equal numbers of outward and inward migrations for Central China with a slightly outward bias (Figure 3B, Supplementary Figure S5). Two introductions of APPV into Central China from Guangdong were supported by the MCC tree with high posterior probability (Figure 2). Meanwhile, Markov rewards for Guangdong (Figure 3C, 321.27) was higher than that for Central China (171.40). Taken together, our results indicated that Guangdong acted as a primary seeding population in China, while Central China established strong epidemic links with multiple regions in this transmission net. In addition, Southwest China set up migration links to five regions of China with a slight inward bias (Figures 3A,B; Supplementary Figure S5, Supplementary Table S6), facilitating the domestic diffusion of APPV.

**Figure 4:**
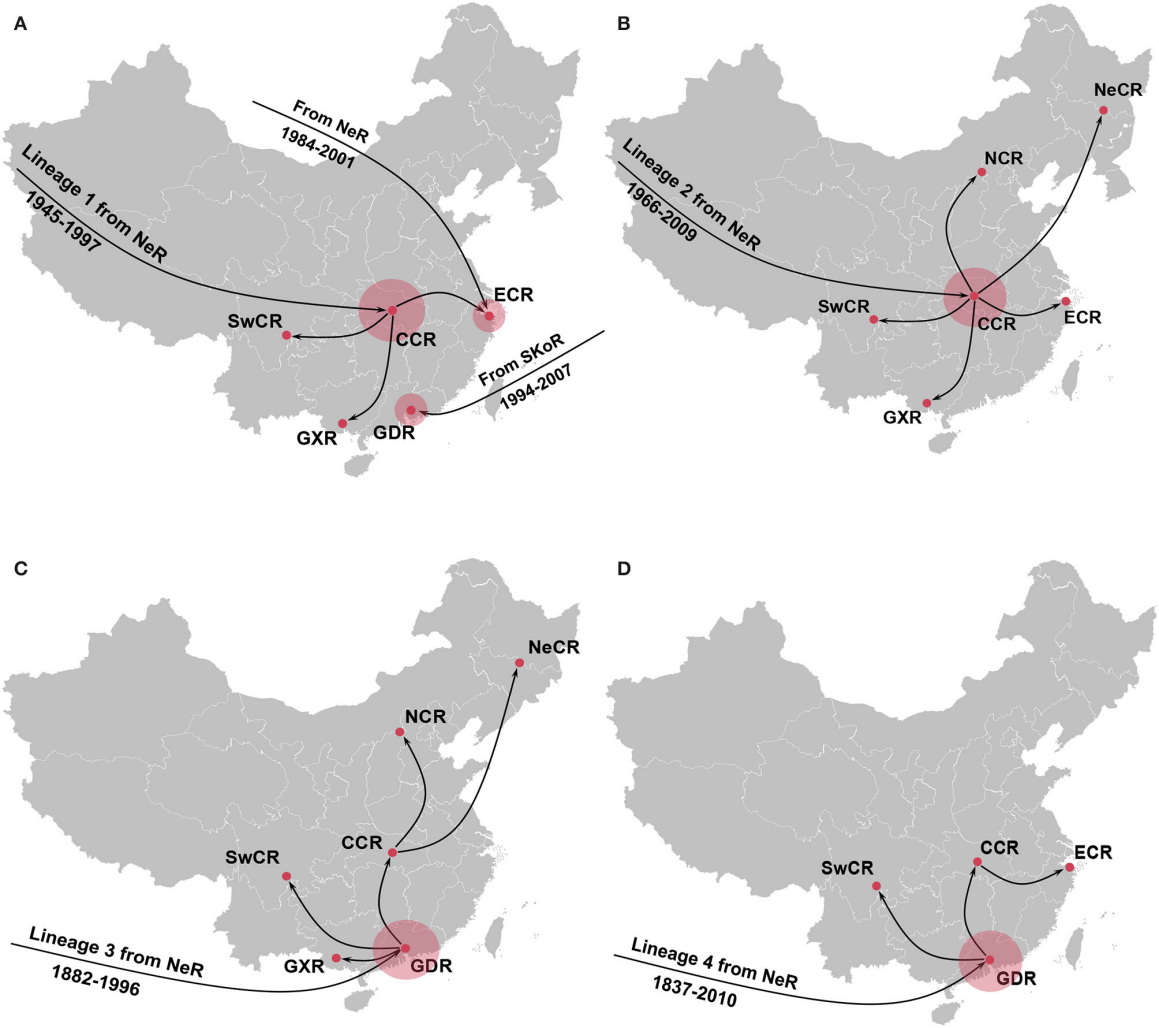
Spatial dynamic of atypical porcine pestivirus lineage 1 **(A)**, lineage 2 **(B)**, lineage 3 **(C)**, and lineage 4 **(D)** in China. Red circles indicate the introduction mainly from the Netherlands seeding transmission pathways in China. NeR, Netherlands Region; SwCR, Southwest China Region; NeCR, Northeast China Region; NCR, North China Region; ECR, East China Region; CCR, Central China Region; GXR, Guangxi Region; GDR, Guangdong Region.

Further phylogeographic analyses showed that APPV lineage 1 was first introduced into Central China from the Netherlands during 1945–1997, and transmitted to East China, Guangxi, and Southwest China (Figure 4A). Although two introductions from the Netherlands to East China (1984–2001) and South Korea to Guangdong (1994–2007) were observed, no migrations from both regions were indicated. Lineage 2 spread from the Netherlands to Central China during 1966–2009. Since then, Central China became the epicenter responsible for the diffusion of APPVs across mainland China (Figure 4B). Lineage 3 migrated from the Netherlands to Guangdong during 1882–1996, and spread to Guangxi, Southwest China, and Central China. Subsequently, Central China became a linker for the diffusion of the virus to North China and Northeast China (Figure 4C). Similar to lineage 3, lineage 4 was introduced into Guangdong during 1837–2010, and spread to Southwest China and Central China. Then, the virus continued to disperse from Central China to East China (Figure 4D). Our results further highlighted that Guangdong and Central China acted as the important regions for the domestic migration of APPV.

### Demographic History of APPV

**Figure 5:**
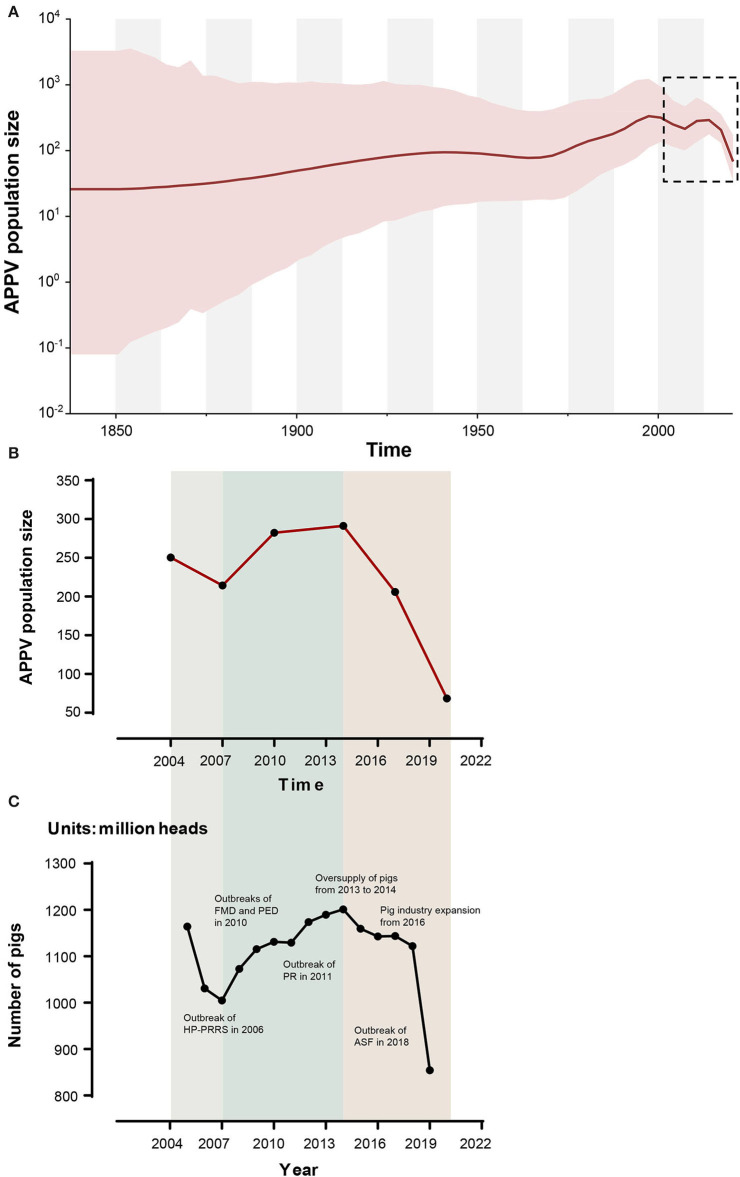
Bayesian skygrid plot of atypical porcine pestivirus (APPV) displaying population size (*y*-axis) through time (*x*-axis). Solid lines represent the median estimates of population size, and the corresponding shaded areas indicate the 95% highest posterior density confidence intervals **(A)**. The population size (median) of APPV between 2004 and 2020 is highlighted by black dotted rectangles and is replotted **(B)**. The dynamic of the number of pigs in China during the past 20 years is shown **(C)**. These data were obtained from the National Bureau of Statistics (https://data.stats.gov.cn/) and EPSDATA (https://www.epsnet.com.cn/) in China. Multiple events during this dynamic are indicated. HP-PRRS, highly pathogenic-porcine reproductive and respiratory syndrome; FMD, foot-and-mouth disease; PED, porcine epidemic diarrhea; PR, porcine pseudorabies; ASF, African swine fever.

Based on the Bayesian skygrid coalescent model, reconstruction of demographic history revealed that the APPV population experienced multiple changes in size (Figure 5). Initially, the population underwent a continuous expansion, followed by a decrease from 1997 to 2007. Then, an upward trend in population size was observed after 2007. From 2014 to 2020, the APPV population experienced a rapid decline in size. Besides, Bayesian skygrid plots individually depicted a waving pattern in the demographic history for lineage 1, which was similar to the pattern of the whole population of APPV (Supplementary Figure S6A). The population size of lineage 1 experienced a persistent expansion until 1995, and a downward trend was observed between 1996 and 2007, followed by an increase before 2013. After 2014, the population size rapidly decreased. For lineage 2, the population maintained a constant size over the past decades (Supplementary Figure S6B). Unlike lineage 2, the populations from both lineage 3 and lineage 4 maintained a relatively stable size with an upward trend through time (Supplementary Figures S6C,D).

### Evolutionary Constraints of APPV Lineages 1–4

**Figure 6:**
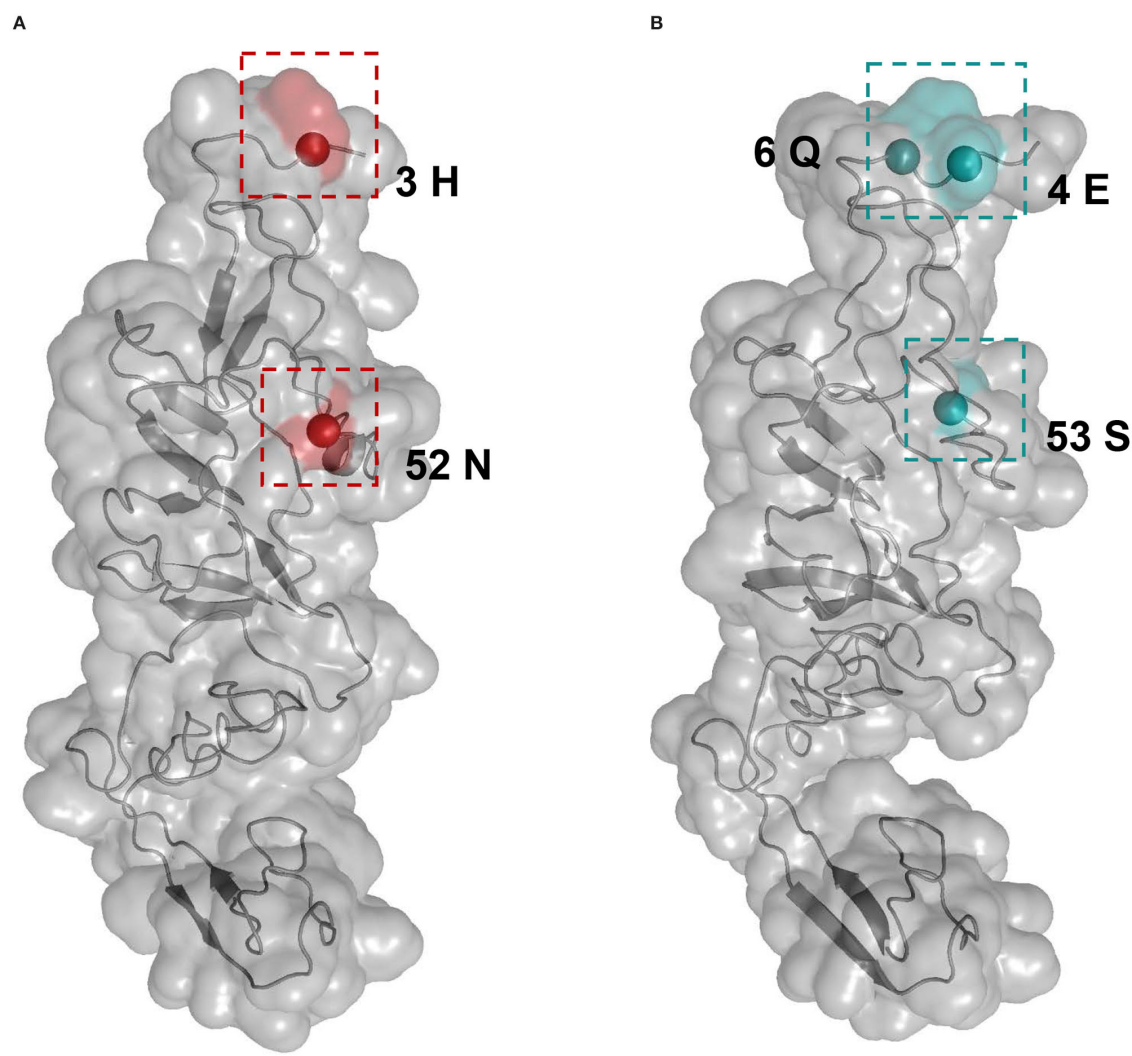
Structural mapping of positively selected sites on the E2 protein. The tertiary structures of E2 monomers were predicted using I-TASSER. **(A)** The model for lineage 1 (Strain: KX929063) was supported with C-score = −1.41 and TM-score = 0.54 ± 0.15. Red colors indicate the residues 3 and 52 from lineage 1. **(B)** The model for lineage 4 (Strain: ON036562) was supported with C-score = −1.45 and TM-score = 0.54 ± 0.15. Blue colors indicate the residues 4, 6, and 53 from lineage 4.

Selective pressure as the ratio of non-synonymous to synonymous (*d*_N_/*d*_S_) substitution rate for each APPV lineage was compared using the two-ratio branch model. The overall *d*_N_/*d*_S_ estimate was 0.0194 for lineage 1, 0.0359 for lineage 2, 0.0145 for lineage 3, and 0.0322 for lineage 4, respectively (Table 2), indicating that the E2 gene is mainly subjected to purifying selection with varying degrees among four lineages. To understand whether individual sites in each lineage were under positive selection, M8 + BEB, MEME, and FEL methods were applied. Consequently, two sites of the E2 gene in APPV lineage 1 and three sites in lineage 4 were under positive selection, which was at least supported by one method (Table 2). We observed complete differences in the positions of these positive sites: positions 3 and 52 for lineage 1 vs. positions 4, 6, and 53 for lineage 4. Comparatively, no positive sites were found in APPV lineages 2 and 3. To visualize these positively selected sites, the tertiary structures of E2 monomers were predicted using I-TASSER. For lineage 1 (Strain: KX929063), the residues 3 and 52, located at the N-terminal loops, were exposed on the protein surface (Figure 6A), of which the former showed the highest variability among lineages (Supplementary Table S7). Likewise, the residues 4, 6, and 53 in lineage 4 (Strain: ON036562) were located at the tips of surface-exposed loops (Figure 6B).

**Table 2:** Comparison of selection pressures of APPV E2 gene for lineages 1–4.

**Lineage**	***Branch d_N_/d_S_* by EasyCodeML**	**Positive selection pressure**					
		**Site** ^**a**^	**MEME**		**FEL**		**M8** **+** **BEB**
			***ω*** **+**	***P*** **value**	***d_N_*/d_S_**	***P*** **value**	***Post. Pro***
Lineage 1	0.0194	3	Inf^b^	0.04*	Inf	0.03*	0.78
		52	3.21	0.27	0.91	0.87	0.95*
Lineage 2	0.0359	None^c^
Lineage 3	0.0145	None
Lineage 4	0.0322	4	Inf	0.04*	Inf	0.03*	1.00**
		6	Inf	0.07	Inf	0.06	0.98*
		53	1.50	0.67	0.63	0.63	0.99**

*Lineage d_N_/d_S_ (ω) was calculated using two-ratio branch model in EasyCodeML. Positively selected sites yielded by MEME and FEL methods in Datamonkey and M8 + BEB method in EasyCodeML.*

*d_N_/d_S_ or ω+, non-synonymous/synonymous; BEB, Bayes empirical Bayes; MEME, mixed effects model of evolution; FEL, fixed effects likelihood; Post. Pro, posterior probability. The sites with statistical significance are indicated by bold values.*

*^a^Site, corresponding to amino acid position of E2 gene.*

*^b^Inf, the value of ω+ or d_N_/d_S_ equals infinity.*

*^c^None, no positive sites were detected.*

^*^Site with one aterisk indicates statistically significant with posterior probability of ≥0.95 or P-value <0.05.

^**^Site with two aterisks indicate statistically significant with posterior probability of ≥0.99 or P-value <0.01.

## Discussion

Pestiviruses are highly mutated RNA viruses, which cause economically relevant diseases in swine, cattle, sheep, and goats. In the last decades, multiple novel pestiviruses have emerged in livestock animals (Smith et al., 2017). The recent identification of a highly distinct pestivirus in pigs, termed APPV, was of particular interest as further discoveries provided evidence for an association of APPV infections with CT type A-II in newborn piglets (Hause et al., 2015; Arruda et al., 2016; De Groof et al., 2016; Postel et al., 2016). In fact, CT syndrome in piglets was first described nearly 100 years ago, yet most cases were attributed to an unidentified virus until 2015 when APPV was discovered using next-generation sequencing technology (Hause et al., 2015). In this study, we aim to investigate the evolutionary history of APPVs by analyzing E2 data from mainland China along with data from other countries. Our Bayesian phylogenetic analyses placed the most recent common ancestor of APPV in 1886 (95% confidence interval: 1837–1924) CE, somewhat earlier than that of the documented emergence of CT disease (1922 CE), implying that APPV is likely not an emerging virus (Arruda et al., 2016).

Given that China has the largest swine population and all four APPV lineages circulate in China, it may have been expected that China would be the source of APPV diversity for other countries in this study. However, since 1886 CE, only two migrations of APPV from China to South Korea were observed, compared to six introductions of Netherlands origin into China, Switzerland, the USA, and Germany. Our phylogenetic analyses placed the root of APPVs in the Netherlands with developed livestock husbandry, providing evidence for the main exporters of APPVs into other countries, which was further supported by the phylogeographic results (Figure 3, State Counts and Markov rewards). Due to the dietary preference for pork, international trade of live swine in China from Europe and America is routine for the consumption of end-stage pork production or for the requirement of breeding pigs for genetic improvement of reproduction or growth traits. We speculated that the introduction of APPV into China from the Netherlands is likely through the transport of live swine rather than airborne spread. This transmission pattern has been confirmed in a study about the global migration dynamic of influenza A viruses in swine (swIAV), demonstrating the importance of the asymmetrical nature of global live swine trade on the global evolution of swIAVs (Nelson et al., 2015).

Our Bayesian phylogeographic analyses revealed Guangdong as a major source of APPV epidemics in China and Central China as a minor source. Strong support for epidemic links between Central China/Southwest China and multiple regions in China was observed. These three regions, covering the main pig-farming provinces of China as expected (such as Sichuan province in Southwest China, Hubei/Henan/Hunan provinces in Central China, and Guangdong province in South China), construct a transmission net of APPVs, which facilitates the diffusion of viruses in China. High swine population density, frequent transport of live swine or pork productions across regions, and suitable climate condition for APPV transmission could explain this migration hierarchy of APPV in China. Additionally, with the implementation of a strict environmental policy since 2000, the pig industry in China has been transferred from South China to Central China and North China presenting a “south to north” movement tendency. Actually, this may accelerate the dispersal of APPVs in China. Interestingly, we found that the “Chinese lineages” (lineage 3 and lineage 4), only detected in China, spread northward from Guangdong Region along this movement route of the pig industry, revealing an important role of pig industry policy in APPV transmission.

Reconstruction of APPV demographic history from temporal phylogenies showed that the virus experienced two rounds of population changes from expansion to shrinkage through time (Figure 5, Supplementary Table S8). Unexpectedly, the demographic pattern of APPV coincided well with the dynamic of the number of pigs in China during the past 20 years. Interestingly, we found multiple events in the key nodes during this dynamic (Figure 5): (1) Outbreak of HP-PRRS (highly pathogenic-porcine reproductive and respiratory syndrome) in 2006 caused a sharp decline in the number of pigs during 2005–2007 (Tian et al., 2007); (2) outbreaks of FMD type O (foot-and-mouth disease) and PED (porcine epidemic diarrhea) in 2010 and outbreak of PR (porcine pseudorabies) in 2011, leading to an overall slowdown in the growth of the number of pigs during 2010–2011 (Li et al., 2012; Valdazo-González et al., 2013; Yu et al., 2014); (3) oversupply of pigs on the market from 2013 to 2014, accelerating the elimination of sows/gilts and inducing a decrease in the number of pigs after 2014; (4) overall expansion of pig industry in China from 2016 contributing to a recovery of pig industry after 2016; and (5) outbreak of ASF (African Swine Fever) in 2018, resulting in a rapid decline of the number of pigs after 2018 (Zhou et al., 2018). This scenario suggested a direct impact of the number of pigs on the population size of APPV. It is considered that the outbreak of major diseases in pigs and human activities related to sow/gilt population changes could influence the population dynamic of APPV. Additionally, we observed that the population size in lineages 2, 3, and 4 maintained relatively stable over the past decades, which was not affected by the changes in the number of pigs in China, suggesting that APPV lineages 2, 3, and 4 might be persistently maintained in China since its initial introduction.

Although the majority of E2 sequences were sequenced since 2016, the phylogenies suggested long-term circulation of APPVs worldwide and the formation of four lineages. Selection pressure analyses showed that different lineages were subject to varying degrees of purification selection. Two positively selected sites in lineage 1 and three sites in lineage 4 were determined with differences in the positions. Interestingly, all positive sites were located in the surface-exposed N-terminal loops. Considering that the N-terminal portion of the pestivirus E2 gene generally contains an antigenically hypervariable region (van Rijn et al., 1994; Chang et al., 2010), and the polymorphisms of positive sites among APPV lineages were observed (Supplementary Table S7), we assumed that positive selection as a driving force may play a role in the evolution of APPV and be likely responsible for the antigenic variation of different lineages. We observed that the mean rate of the APPV E2 gene was 1.22 × 10^−3^ substitutions/site/year, which was lower than that estimated for another important porcine pestivirus, for example, classical swine fever virus (CSFV) (An et al., 2018). It is likely that the long-term and widespread use of vaccines accelerates the evolution of CSFVs. This can provide an explanation for the lower rate of the E2 gene in APPV possibly due to the weaker immune selection (no vaccine is available for APPV). We found a strong association between phylogeny and geographic trait (Table 1), revealing geography-driven adaptation as an important factor for the diversification of APPV. Actually, this scenario has been supposed in a recent study from Japan, in which APPVs may independently evolve in the Japanese pig population (Kasahara-Kamiie et al., 2021). In addition, we failed to find recombination signals in the E2 region in our study, although previous works have revealed the presence of recombination events in the non-structural gene regions (Guo et al., 2020). A plausible explanation is that the E2 gene is not a hotspot for recombination. Further studies are needed to identify the role of E2 recombination in the evolution of APPV.

In summary, our study provided comprehensive insights into the spatiotemporally evolutionary dynamic of APPV based on the available E2 sequences, revealing that APPV has likely been circulating worldwide for over a century. The Netherlands potentially acted as the main source for the epidemics in other countries, from which multiple introductions into China occurred during 1837–2010. While in China, Guangdong acted as a primary seeding population and constructed a transmission net of APPV together with Central China and Southwest China as epidemic linkers. This dispersal pattern is likely to be related to the frequent transport of live swine or pork productions across regions in China. Interestingly, we found that the migration pathways of lineage 3 and lineage 4 in China traced a “south to north” transfer tendency of the domestic pig industry. Unexpectedly, the demographic dynamic of APPV coincided well with the changes in the number of pigs in mainland China in the past decades, which was possibly affected by the outbreak of major diseases in pigs and human activities related to sow/gilt population changes. In addition, selection constraints and geography-driven adaptation might play important roles in the diversification of APPV. Continued surveillance for APPV is clearly required to determine their long-term evolutionary dynamic.

However, we observed long branch lengths in the inferred phylogenies in our study (Figure 2). This may conceal additional spatial migrations during the elapsed time. The lack of historical APPV genomic data together with long branches in phylogenies make it difficult to clearly resolve the evolutionary history of APPV in China or worldwide. We acknowledge that the evolutionary dynamic of APPV presented here has been inferred only from the E2 dataset, on which the influence of sampling bias is unavoidable. Although heterogeneous sampling can bias and affect the output of some phylodynamic and phylogeographic methods, the general patterns in the spatiotemporal dynamic of viruses could be still inferred from a dataset with smaller samples from individual locations (Lemey et al., 2020; Kalkauskas et al., 2021). We hold that large full-genome datasets with a broader temporal-spatial window will contribute to clarifying the evolutionary history of APPV in the future.

## Data Availability Statement

The datasets presented in this study can be found in online repositories. The names of the repository/repositories and accession number(s) can be found in the article/Supplementary Material.

## Author Contributions

HM conceived and designed the experiments, performed the analyses of data, and wrote the article. WL, MZ, and QH provided pieces of advice on this paper framework and helped in polishing the language. HM, ZY, and LL collected the samples. AG provided help in polishing the language of this article. All authors agreed on the manuscript before review.

## Funding

This work was supported by China Agriculture Research System (No. CARS-35).

## Conflict of Interest

The authors declare that the research was conducted in the absence of any commercial or financial relationships that could be construed as a potential conflict of interest.

## Publisher's Note

All claims expressed in this article are solely those of the authors and do not necessarily represent those of their affiliated organizations, or those of the publisher, the editors and the reviewers. Any product that may be evaluated in this article, or claim that may be made by its manufacturer, is not guaranteed or endorsed by the publisher.
