# Supplementary figures and images for: The Diversity and Spatiotemporally Evolutionary Dynamic of Atypical Porcine Pestivirus in China
Source: Front Microbiol. 2022 Jun 24;13:937918. doi: 10.3389/fmicb.2022.937918 (PMC9263985; doi:10.3389/fmicb.2022.937918)

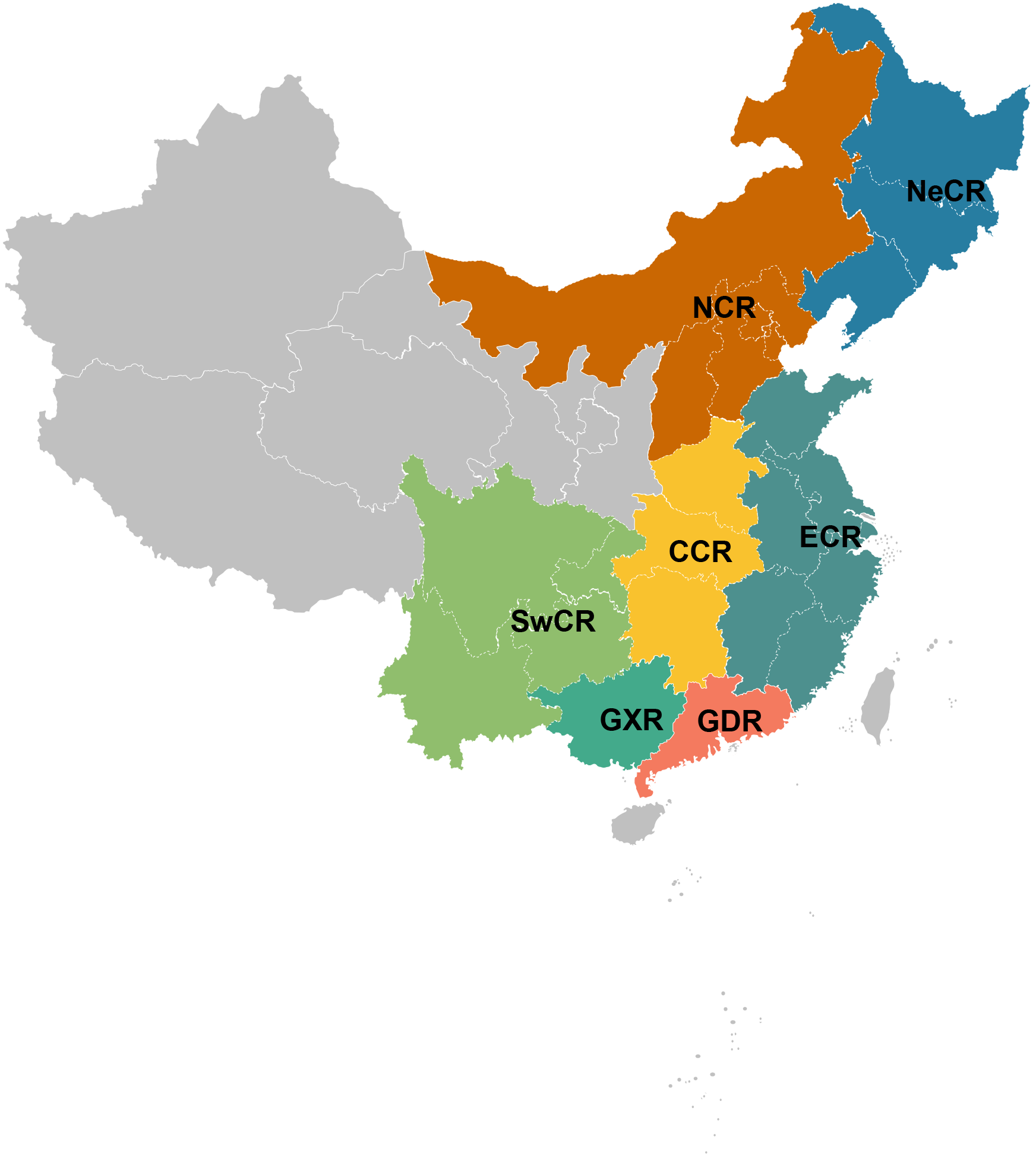

Supplement: Supplementary file 9 [file Image_1.TIF]

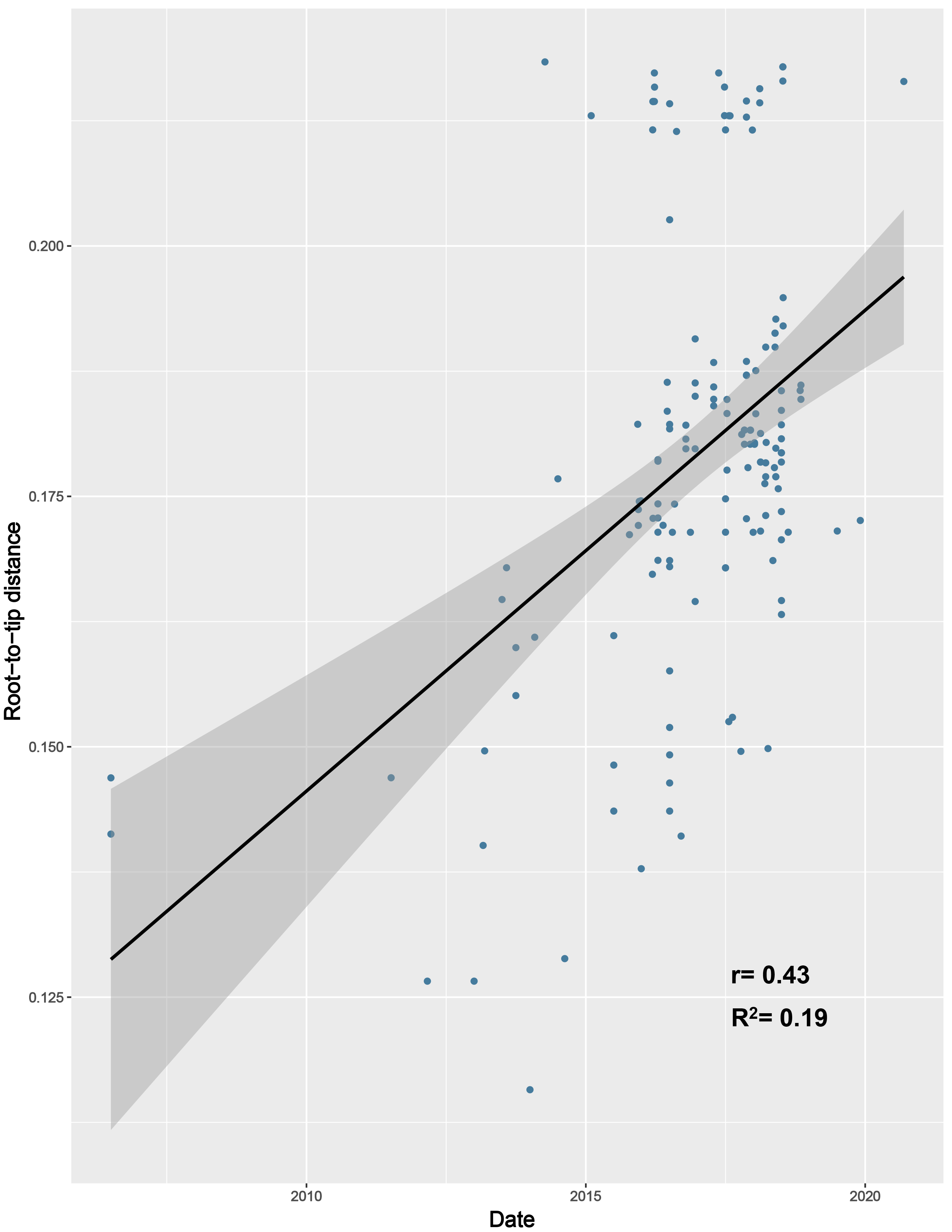

Supplement: Supplementary file 10 [file Image_2.TIF]

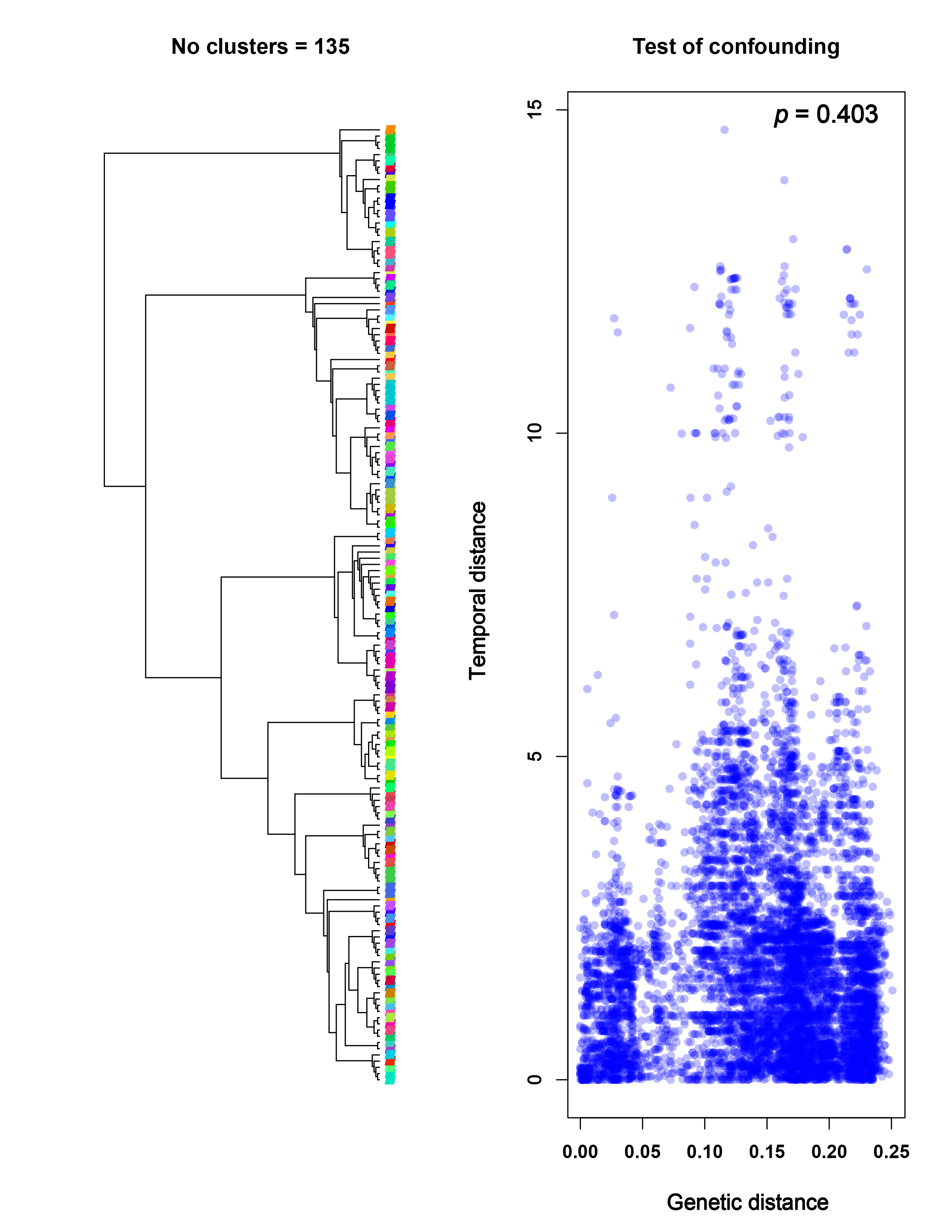

Supplement: Supplementary file 11 [file Image_3.TIF]

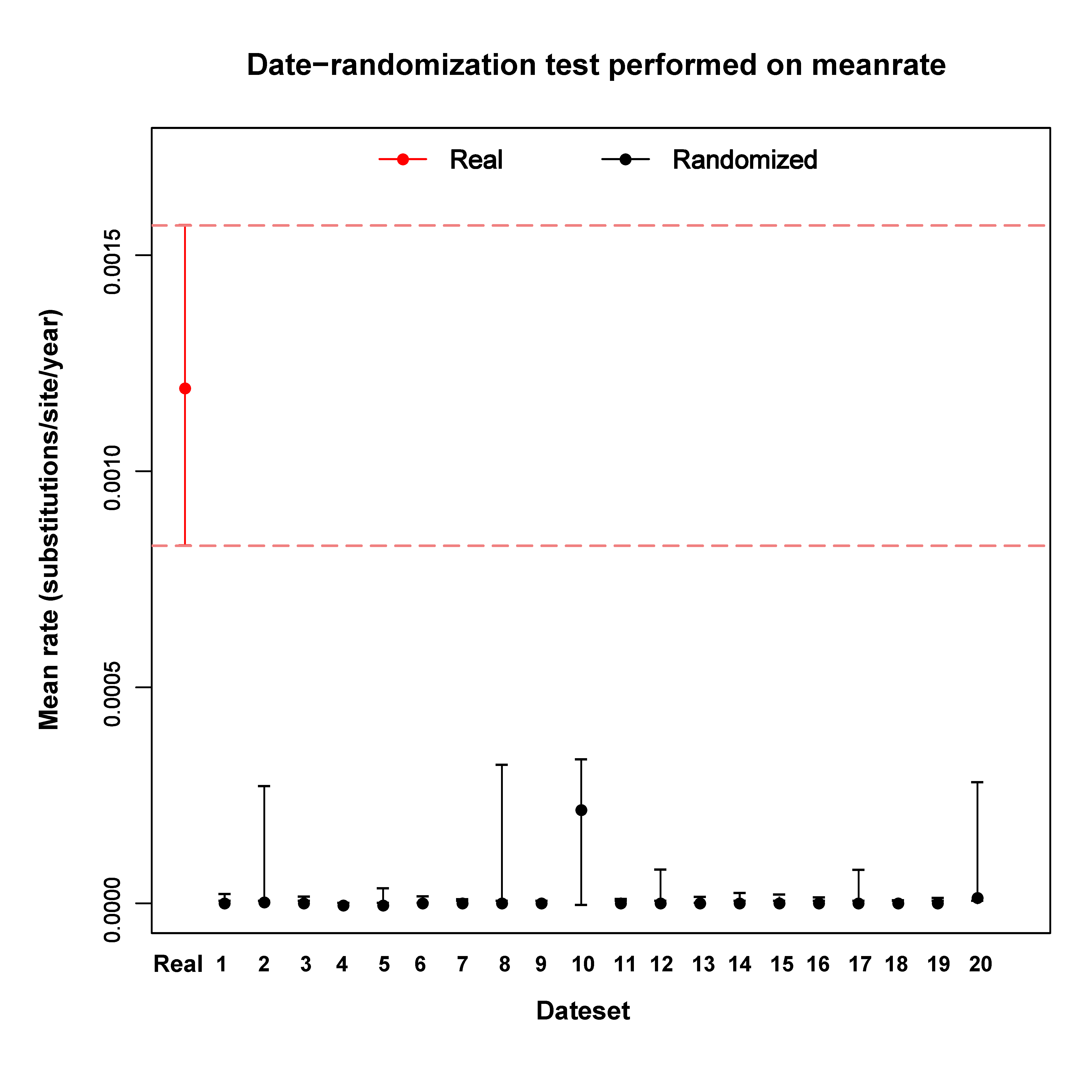

Supplement: Supplementary file 12 [file Image_4.TIFF]

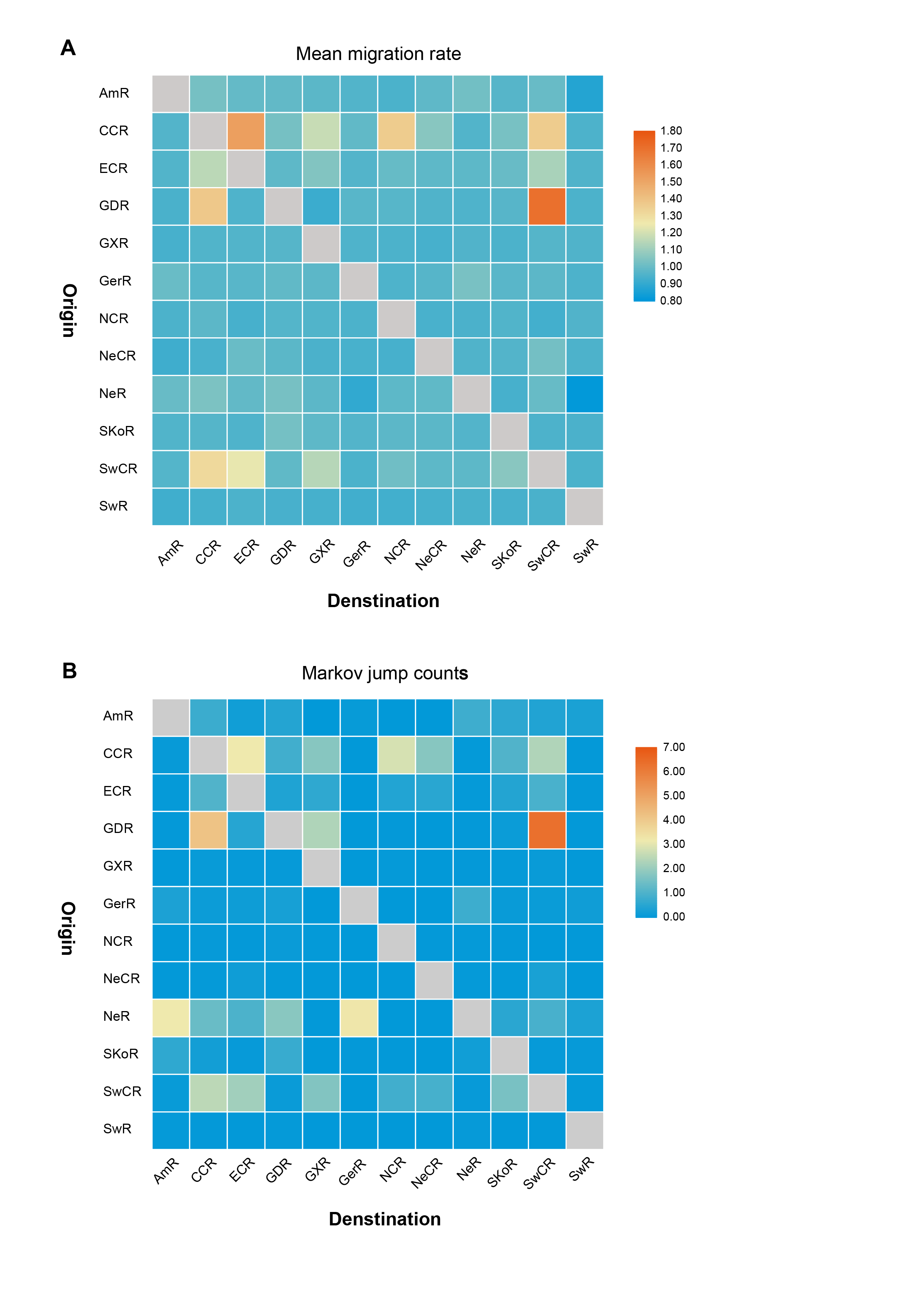

Supplement: Supplementary file 13 [file Image_5.TIF]

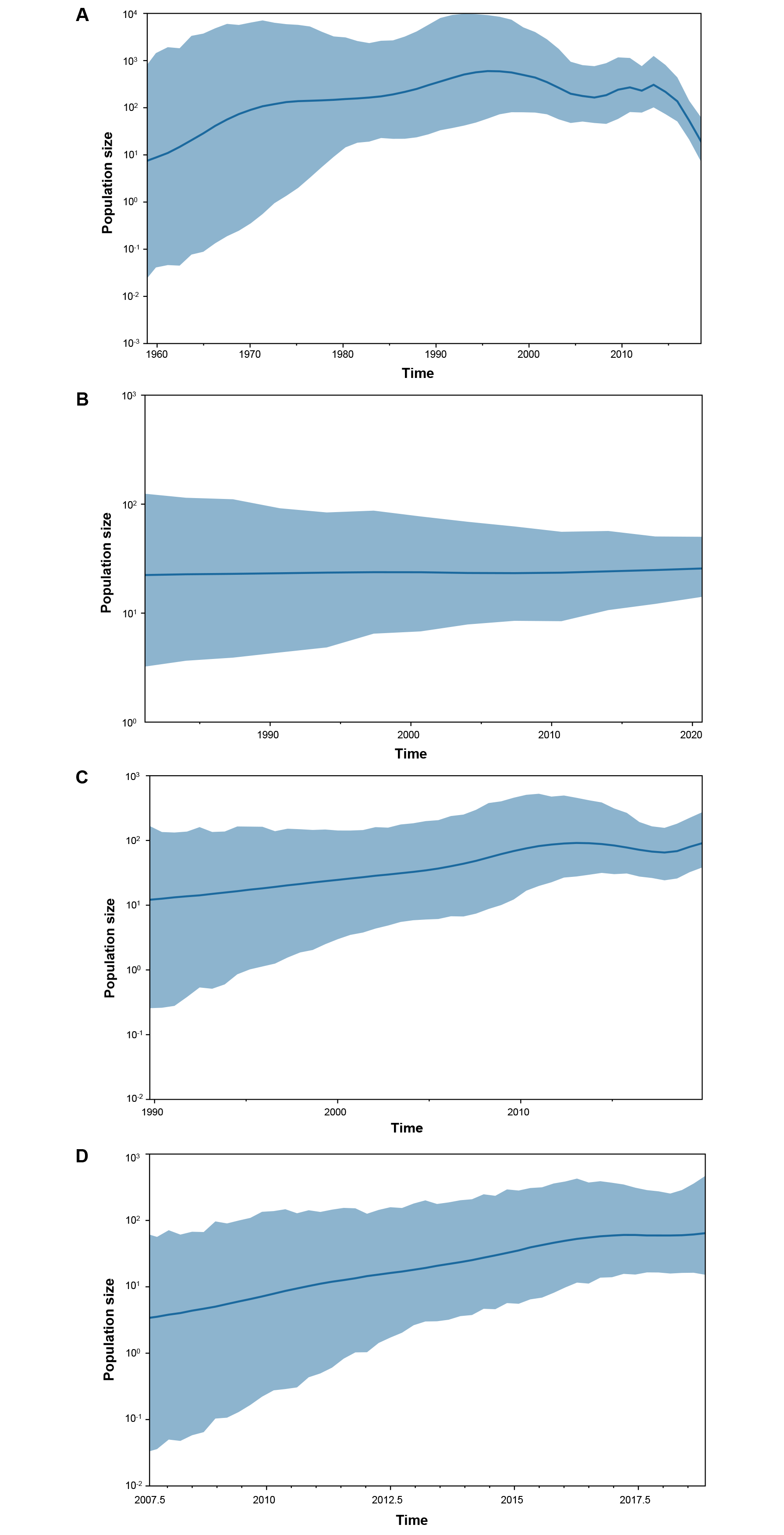

Supplement: Supplementary file 14 [file Image_6.TIF]
